# The complete mitochondrial genome of the *Sideridis albicosta* (Lepidoptera: Noctuoidea: Noctuidae)

**DOI:** 10.1080/23802359.2018.1462114

**Published:** 2018-04-23

**Authors:** Shun Yao, Zi-Hui Wang, Xiao-Yue Zhu, Jia-Sheng Hao

**Affiliations:** Laboratory of Molecular Evolution and Biodiversity, College of Life Sciences, Anhui Normal University, Wuhu, PR China

**Keywords:** Complete mitochondrial genome, phylogeny, Noctuoidea, *Sideridis albicosta*

## Abstract

The complete mitochondrial genome (mitogenome) of *Sideridis albicosta* is described in this study. The circular molecule is 15,320 bp in length and contains 13 protein-coding genes (PCGs), two rRNA genes, 22 tRNA genes, and one AT-rich region. Thirteen PCGs is 11,252 bp in total, encoding 3723 amino acids. All PCGs start with ATN, except for COI gene starting with CGA; 10 of the 13 PCGs use the typical stop codon TAA, whereas COI, COII, and ND4 stop with a single T. The *rrnL* and *rrnS* genes are 1377 bp and 783 bp in length, respectively. The 328 bp AT-rich region contains several structures characteristic of the lepidopterans. New phylogenetic analyses upon mitogenomics would provide us further insights on the taxonomy and phylogeny of Noctuoidea.

The superfamily Noctuoidea of Lepidoptera comprises about 20,000 species distributed nearly all around the world. However, up to date, their taxonomy and phylogeny are still standing as a controversial issue, and need to be further elucidated (Mitchell et al. 2006; Zahiri et al. 2013; Regier et al. 2017). In recent decades, the insect mitogenomes have been widely used as an informative molecular marker for phylogenetic and population genetic studies at various hierarchical levels, due to their unique features (Salvato et al. 2008; Yin et al. 2010).

In this study, we newly determined the complete mitochondrial genome of *Sideridis albicosta*, a member commonly found in China in the family Noctuidae of Noctuoidea. Adult individuals of *S. albicosta* were collected at Xuancheng, Anhui province, China on September 2017. After morphological identification, the specimens were stored at −70 °C in our laboratory (Lab of Molecular Phylogeny and Evolution, Anhui Normal University).Total genomic DNA was extracted from the thorax muscle of a single individual using the Sangon Animal genome DNA Extraction Kit (Shanghai, China). Whole mitochondrial genome was amplified with 16 pairs of primers and sequenced by protocols that are routinely used in our laboratory (Shi et al. 2015). The resultant reads were assembled and annotated using the BioEdit 7.0 (Hall 1999) and MEGA 6.0 software (Tamura et al. 2013).

The whole mitochondrial genome is a circular molecule of 15,320 bp in size, with an AT bias of 80.3%, and contains 13 protein-coding genes (PCGs), 22 tRNA genes, and two rRNA genes and a non-coding AT-rich region (D-loop) (GenBank Accession No. MH027985). The gene arrangement and orientation are identical with other lepidopteran species (Wu et al. 2013; Hou et al. 2014). Thirteen PCGs are 11,252 bp in total, encoding 3723 amino acids. All tRNAs harbour the cloverleaf secondary structures predicted by tRNAscan-SE (Schattner et al. 2005) except for the tRNA^-Ser^(AGN) lacking the DHU-arm. All PCGs start with a typical ATN codon, except COI which is started with CGA; 10 PCGs (COIII, ND1, ND2, ND3, ND4L, ND5, ND6, Cytb ATP6, and ATP8) stop with usual codon TAA, while three PCGs (COI, COII, and ND4) stop with incomplete codon T. The *rrnS* and *rrnL* genes are 783 bp and 1377 bp in length, respectively. The AT-rich region is 328 bp in size, harbouring some structures characteristic of lepidopterans.

Using two Geometridae species as the outgroups, the phylogeny of 44 Noctuoidea species, including the *S. albicosta*, was reconstructed based on nucleotide sequence data of 13 PCGs with the maximum-likelihood (ML) and Bayesian inference (BI) methods through RAxML (version 7.2.6) and MrBayes (version 3.2.2). The resultant ML and BI trees distinctly showed that the *S. albicosta* constituted a monophyletic group with other 19 Noctuidae species; additionally, the two trees obviously indicated that all the involved species constituted four major clades: the first was the *Clostera anachoreta*; the second was the other two species of Notodontidae; the third included the Arctiidae and Lymantridae; and the fourth was made up of the Nolidae and Noctuidae (Figure 1).

**Figure 1.**
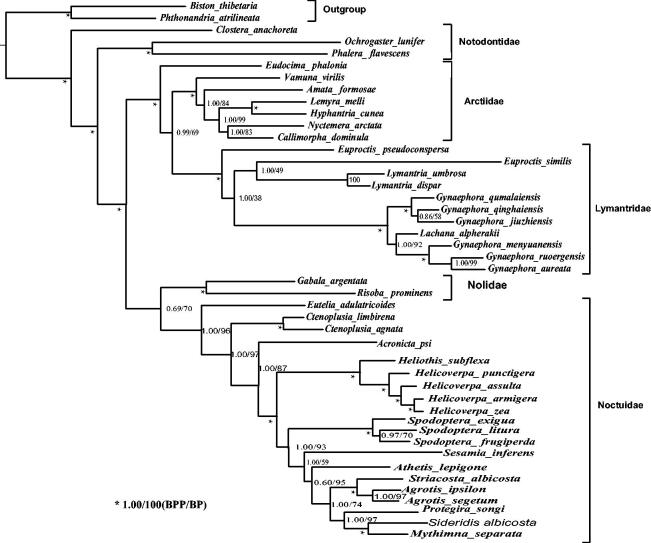
Bayesian inference (BI) and maximum likelihood (ML) phylogenetic trees inferred from the nucleotide sequence data of mitogenomic 13 PCGs.
